# Influence of environmental change, harvest exposure, and human disturbance on population trends of greater sage-grouse

**DOI:** 10.1371/journal.pone.0257198

**Published:** 2021-09-24

**Authors:** Jonathan B. Dinkins, Kirstie J. Lawson, Jeffrey L. Beck

**Affiliations:** 1 Department of Ecosystem Science and Management, University of Wyoming, Laramie, Wyoming, United States of America; 2 Department of Animal and Rangeland Sciences, Oregon State University, Corvallis, Oregon, United States of America; U.S. Geological Survey, UNITED STATES

## Abstract

Hunter harvest of greater sage-grouse (*Centrocercus urophasianus*; hereafter “sage-grouse”) has been regulated by wildlife agencies during most of the past century. Hunting season regulations were maintained with the intention of providing sustainable hunting opportunities. Sage-grouse populations oscillate over time, and population growth can be influenced by seasonal weather and habitat disturbance. From 1995–2013, we compared sage-grouse lek trends from 22 relatively distinct sage-grouse population segments in 9 western U.S. states and 2 Canadian provinces. We stratified these populations into 3 broad categories (non-hunted [*n* = 8], continuously hunted [*n* = 10], and hunting season discontinued between 1996–2003 [*n* = 4]) with 8 different regulation histories to evaluate the potential impact of harvest on sage-grouse populations. Concomitantly, we assessed the effects of proportion burned, forested and cropland habitat; winter, spring, and summer precipitation; and human population, road, and oil and gas well densities on initial and time-varying lek counts. Density-dependent models fit lek trend data best for all regulation histories. In general, higher proportions of burnt, forested, and cropland habitat; and greater human population and oil and gas well densities were associated with lower equilibrium abundance (*K*). We found mixed results regarding the effect of hunting regulations on instantaneous growth rate (*r*). The cessation of harvest from 1996–2001 in approximately half of the largest sage-grouse population in our analysis was associated with higher *r*. Continuously harvested sage-grouse populations with permit hunting seasons had higher *r* during years with higher proportion of area exposed to permitted hunting rather than general upland game seasons. However, more liberal hunting regulations were positively associated with higher *r* in populations continuously harvested under general upland game hunts. Our results suggest that discontinuing harvest in the largest population resulted in greater population growth rates; however, this was not consistently the case for smaller populations. To no surprise, not all sage-grouse populations were influenced by the same environmental change or human disturbance factors. Our results will assist managers to understand factors associated with *K*, which provides the best targets for conservation efforts.

## Introduction

State and provincial wildlife management agencies in the United States and Canada have been tasked with maintaining viable wildlife populations. These agencies categorize population status and trends of numerous wildlife species and designate appropriate conservation actions for species of conservation concern. In addition to maintaining viable wildlife populations, wildlife agencies are concurrently mandated to manage and provide the public with hunting seasons for game species [1]. For example, wildlife management agencies simultaneously monitor population trends, propose and implement conservation actions, and regulate hunting seasons for greater sage-grouse (*Centrocercus urophasianus*: hereafter ‘sage-grouse’). Meanwhile, the distribution and abundance of sage-grouse in western North America has continued to decline over the last century [2–4]. Continued loss of sagebrush (*Artemisia* spp.) habitat has been identified as the largest threat to sage-grouse population persistence [5–9]. These issues have prompted multiple petitions to the U.S. Fish and Wildlife Service (USFWS) to list sage-grouse as threatened or endangered throughout its range in the United States [10], and an endangered listing under the Canadian Species at Risk Act [11]. There have been many concerns from the public about the continued human hunting of sage-grouse from both biological and sociological perspectives.

Habitat fragmentation, loss, and degradation have been directly attributed to reduced long-term demographic rates and population decline [5, 9, 12, 13]. Population viability of sage-grouse has been linked to adult female survival and breeding success [14, 15]. Thus, conservation activities and policies have been focused on breeding habitat [9]. Higher quantity and quality of breeding habitat has been positively connected to the number of male sage-grouse in attendance at breeding display grounds (leks; [8, 9, 12, 13]. This suggests the condition of breeding habitat is positively related to multiple demographic rates (adult, nest, and chick survival), which in turn have been connected to population growth and carrying capacity. Avoidance of forests and woodlands by sage-grouse has been well documented [9, 16–19], and lower sage-grouse lek counts have been associated with proximity to trees [8, 9]. Forests and woodlands increase the risk of predation to sage-grouse [17, 20–22] with female sage-grouse survival during the breeding season and summer lower near trees [22]. Fire occurrence and extent have increased in sagebrush habitat since the mid-1980s, which has been associated with sage-grouse lek declines in the Great Basin [13]. Similar to changing environmental factors, increases in human infrastructure and disturbance have been negatively associated with sage-grouse lek counts [9, 12, 23–25] and lek persistence [26, 27]. In addition, persistence of sage-grouse populations, as quantified by active or extirpated leks, has been positively related to lower human population density, less cultivated cropland, and fewer severe droughts [6]; less fire [26]; and absence of encroaching pinyon–juniper [21].

Hunter harvest has been suggested as a potential mechanism contributing to dampened sage-grouse population size through potential additive mortality [14, 28–31]. The 2010 USFWS listing decision indicated uncertainty about whether harvest had been compensatory or additive to sage-grouse populations [32]. Hunter harvest of sage-grouse has the potential to decrease survival rates and recruitment of young into the breeding population. Connelly et al. [30] analyzed three levels of harvest exposure (no harvest, 1-bird daily bag limit [2-bird possession limit] with 9-day hunting season, and 2-bird daily bag limit [4-bird possession limit] with 23-day hunting season) in southern Idaho. Their results demonstrated that sage-grouse hunting can negatively influence spring lek counts, particularly where habitat quality is low [30]. For these reasons, wildlife agencies have implemented increasingly more conservative season regulations since the mid-1990’s [2, 33, unpublished data]. These hunting regulations were intended to maintain harvest at sustainable levels. Some studies suggest that harvest mortality was compensatory [34–36], and sage-grouse populations in Washington (Moses Coulee and Yakima Training Center sage-grouse populations) did not increase following permanent hunting season closure after 1987 in Washington [33]. Hunting regulation restrictions were also based on recommendations that hunter take of sage-grouse should not exceed 10% [29, 37] or 5% [33] of the fall population. However, the fall population size has typically been unknown for sage-grouse.

Identification of environmental factors and human activities associated with poor population growth and/or lower carrying capacity is essential for wildlife agencies to achieve goals of simultaneously maintaining viable populations and hunting seasons for the public. Confounding to the potential influence of hunter harvest on population growth, sage-grouse populations oscillate over time with population growth influenced by seasonal weather and habitat disturbance [13, 38]. Thus, our study simultaneously assessed environmental (fire, habitat condition [cropland, and tree cover], seasonal precipitation) and human activities (human population density, anthropogenic features [cities, coal mines, communication towers, oil and gas wells, power lines, roads, wind turbines], and hunting regulations) as factors influencing sage-grouse population growth or carrying capacity. The accuracy and spatial influence of actual harvest on sage-grouse and potential effects of density dependence prevented the use of number of birds shot as a covariate to describe population growth. Thus, we used an integrated approach to assess population trends of smaller sage-grouse populations with distinct regulation histories from 1995–2013, while simultaneously evaluating the effects of environmental change and human activities. Our analyses capitalized on assessing harvest exposure variables while also allowing us to compare trends among populations with different regulation histories: 1) hunting season across the duration of the study, 2) hunting seasons only for a few years, and 3) no hunting seasons since 1990 or before.

We predicted anthropogenic development, fire, and forested habitat would be negatively related to lek counts and trends via changes in equilibrium abundance (carrying capacity [*K*]) across time, which represented change in habitat quality relative to nesting and brood-rearing habitat near leks for sage-grouse. However, breeding habitat is used by sage-grouse year-round [9], therefore, would also be related to adult survival rates. Finally, we predicted positive precipitation and negative harvest exposure influences on population growth via annual reproductive success and adult survival, respectively.

## Study area

**Fig 1:**
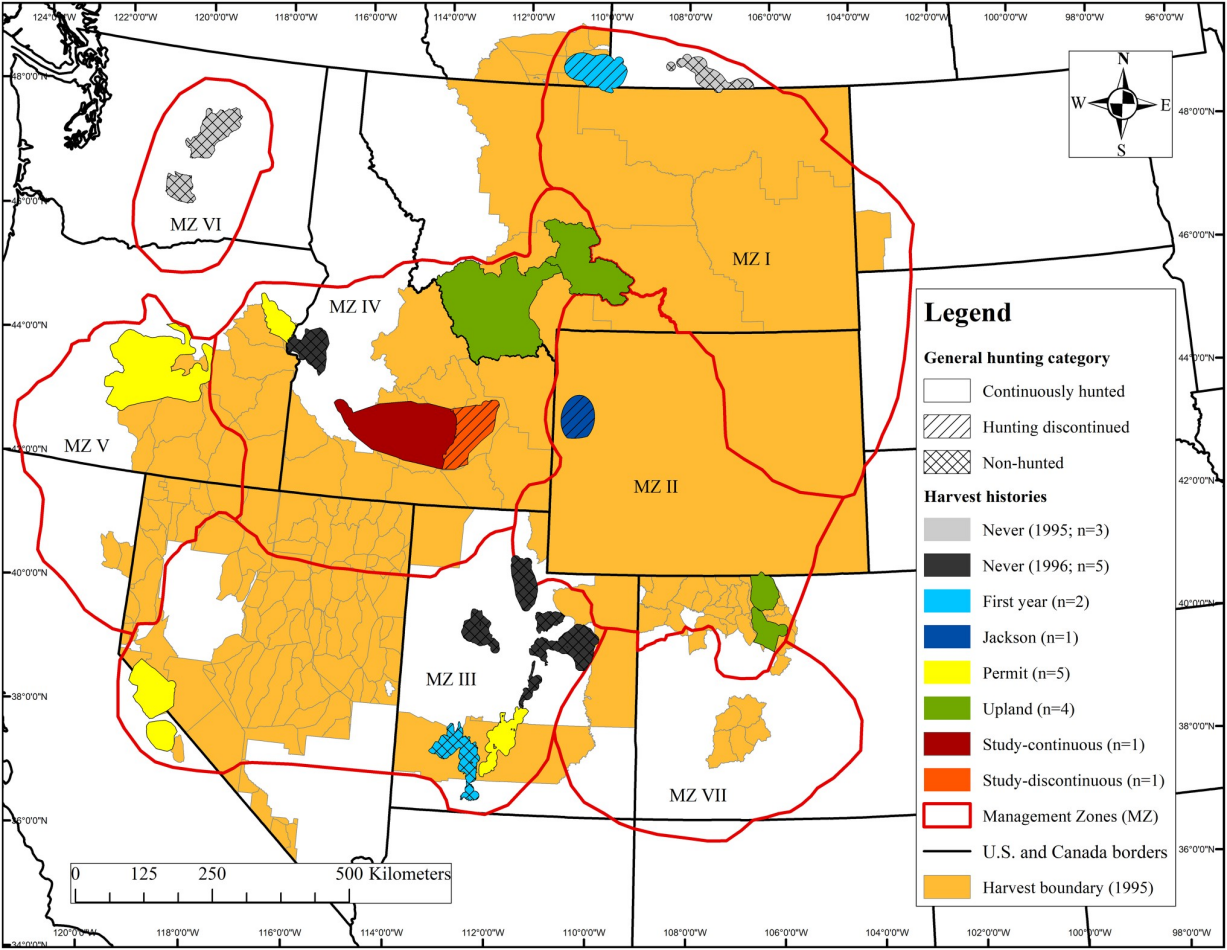
Map of 21 relatively distinct sage-grouse populations stratified into 8 regulation histories. Orange background portrays legal hunting season boundaries in 1995. Light green delineates the presumed distribution of sage-grouse, and multi-colored polygons indicate 8 km buffers around active sage-grouse leks within these populations as of 1995. Lek data were collected by states and provinces throughout the western United States and southern Alberta and Saskatchewan Canada from 1995–2013.

Our study consisted of 21 relatively distinct sage-grouse populations from 9 western states in the United States and 2 Canadian provinces (Fig 1, Table 1). These populations were delineated by USFWS [10]. We then stratified them into 8 regulation histories, which were defined by the years when hunting seasons for sage-grouse occurred within each population during 1995–2013 (Table 1). Populations were located in sagebrush steppe ecosystems with expansive big (*Artemisia tridentata* ssp.) or silver (*A*. *cana*) sagebrush; however, sagebrush cover varied among populations.

**Table 1:** Descriptions of sage-grouse populations, Western Association of Fish and Wildlife Agencies Management Zones (MZ), years hunted, and lek sample sizes used in N-mixture models. Regulation histories were stratified by sage-grouse populations with the same years when human harvest of sage-grouse occurred (*n* = 8 regulation histories). The ‘Never’ regulation history was subdivided into SG populations with adequate data for 1995–2013 and 1996–2013. Male count data were collected by states/provinces throughout the western U.S. and southern Alberta and Saskatchewan Canada from 1995–2013.

Regulation history	Sage-grouse populations	MZ	Years hunted	Leks
Never_1995_	Moses Coulee, WA^a^	VI	N/A	14
	Saskatchewan, Canada^b^	I	N/A	7
	Yakima Training Center, WA^a^	VI	N/A	9
				Total = 30
Never_1996_	Northeast Interior, UT^c^	III	N/A	17
	Sanpete, UT^d^	III	N/A	2
	Sheeprock Mountain, UT^e^	III	N/A	4
	Summit/Morgan, UT^f^	III	N/A	3
	Weiser, ID^g^	IV	N/A	13
				Total = 39
First-Year	Alberta, Canada	I	1995	14
	Southwest, UT	III	1995	11
				Total = 25
Jackson	Jackson, WY	II	1995–1999, 2002	Total = 8
Permit	Baker, OR	IV	1995–2013	12
	Central, OR	V	1995–2013	63
	North Mono Lake, CA/NV^h^	III	1995–2013	16
	Parker Mountain, UT^i^	III	1995–2013	22
	South Mono Lake, CA	III	1995–2013	11
				Total = 124
General	Belt, MT	IV	1995–2013	8
	Middle Park, CO	II	1995–2013	15
	North Park, CO	II	1995–2013	44
	Southwest, MT	IV	1995–2013	16
				Total = 83
Study-Continuous	Snake/Salmon/Beaverhead (partial), ID	IV	1995–2013	Total = 59
Study-Discontinuous	Snake/Salmon/Beaverhead (partial), ID	IV	1995, 2002–2013	Total = 32

^a^Last hunting season in fall 1987.

^b^Last hunting season in fall 1950’s.

^c^Last hunting season in fall 1989.

^d^Last hunting season in fall 1990.

^e^Last hunting season in fall 1981.

^f^Last hunting season in fall 1984.

^g^Last hunting season in fall 1984.

^h^Last hunting season in Nevada was fall 1998.

^i^Permit only hunting seasons were implemented in 2000 with an unlimited number of permits with restrictions on number of permits beginning in 2002.

We focused our study on the area within each sage-grouse population where most sage-grouse where likely to occur, which we represented as an 8-km buffer around all sage-grouse lek locations within each population that had a male lek count ≥2 in at least one year 1995–2013 (sage-grouse leks and counts defined in lek data). Our choice of an 8-km buffer was intended to be inclusive of areas where sage-grouse were most likely to be located within sagebrush habitat on an annual basis. This delineation of sage-grouse populations aligned with distances from sage-grouse use locations to leks in the bi-state (along the California and Nevada border) sage-grouse population [39], Utah [40], and Wyoming [41]; 8.4 km from lek locations was also used by Doherty et al. [42] to delineate rangewide sage-grouse habitat and distribution. Our mapping of sage-grouse populations was also consistent with Garton et al. [3] mapping of relatively distinct sage-grouse populations.

## Materials and methods

### Lek data

During spring, male sage-grouse strut at display grounds (leks) with historical fidelity to spatially explicit locations. State and provincial wildlife agencies have maintained counts of male sage-grouse at these locations as an index to monitor change in population trends [2]. We obtained 1995–2013 lek data from wildlife management agencies and the Western Association of Fish and Wildlife Agencies (WAFWA) to assess factors influencing lek trends. These years encompassed the highest quality and consistent sage-grouse lek count data. Lek data consisted of the spatial location of display grounds and annual counts of male sage-grouse. WAFWA provided the peak or highest count of male sage-grouse observed during ≥1 observation per year [3, 4, 43].

Male count data from all wildlife management agencies had missing counts for leks used in our analyses during some years. While missing lek count data across the entire landscape likely has bias related to access and focus on collecting data at leks with more males, we assumed that missing count data among lek locations and years used in our study was relatively random as related to our analyses. Annual lek data within each state and provincial database were recorded under protocols requiring a minimum of 3 visual observations per year to determine a valid peak male count. However, more recent annual lek data within some state databases allowed inclusion of peak male counts from leks counted <3 times in a year. Fedy and Aldridge [43] found that inclusion of peak male counts from leks with <3 observations in a year did not bias lek trend estimates. Lek trend estimates were improved by including more count information temporally and spatially (i.e., fewer years of missing data for individual leks and inclusion of more leks in trend analyses; [43]). We could not determine how many times each lek was counted during each year, because this information was not included in the lek database provided to us by WAFWA.

Trends derived from lek counts are an index rather than a true population count, because only males can be counted reliably [44]. Count methods varied slightly among states and provinces [2, 5]. No state or province significantly changed their lek count protocol since 1995; therefore, lek counts post-1995 can be considered spatiotemporally relative for population trend analyses. We assumed that each state and provincial wildlife agency reported legitimate peak male count data that were representative of a relative population index across time.

Leks considered for our analyses were defined in a similar fashion to Fedy and Aldridge [43] and Nielson et al. [4] with a couple of notable exceptions. First, we only included leks where ≥2 males were counted during at least 1 year from 1995–2013. Second, our analyses focused on evaluating multiple spatial and temporal factors influencing lek trends across time. Newly discovered lek locations could bias trend estimates across time, because they did not have the opportunity to be counted during the early years of our study. Thus, we only included leks that had at least two consecutive counts (i.e., one calculation of change) during each of three timespans (1995–2000, 2001–2006, and 2007–2013).

### Human activity and environmental change variables

We compiled an inclusive set of GIS-derived variables related to human activities and environmental change throughout the 21 relatively distinct sage-grouse populations (S1 Table). All variables included were quantifiable at the same spatial extent among all sage-grouse populations. Human activity variables included distance to a town; human population, major and gravel road, oil and gas well, power line (transmission lines), and wind turbine densities; proportion of habitat as cropland; and hunter harvest exposure (i.e., indices of hunter harvest pressure; S1 Table). Environmental change variables included proportion of habitat burned (fire proportion) or forested, and precipitation (S1 Table). However, environmental variables likely had exogenous inputs from human endeavors. We used ArcMap version 10.2 (Esri, Redlands, CA, USA), Geospatial Modeling Environment version 0.7.3.0 (http://www.spatialecology.com/gme), and the ‘raster’ package version 2.3–40 in R version 3.0.2 (R Development Core Team 2015) to calculate and extract these variables relative to sage-grouse leks. All explanatory variables were quantified as average value within 8 km of a sage-grouse lek from circular moving windows with the exception of precipitation and harvest exposure variables. Pixel size and data source of each variable are described in S1 Table.

Our calculation of explanatory variables with potential to directly influence breeding habitat was restricted to within 8 km of individual leks (S1 Table), because (1) the spatial position of male sage-grouse forming leks near nesting and brood-rearing habitat has been connected to where males are likely to encounter females [31], (2) sage-grouse lek locations are predominantly positioned in areas surrounded by nesting and brood-rearing habitat [39, 45–49], and (3) most females nest within 8 km of a lek [40, 47]. We quantified precipitation variables as total accumulation within 10 km of each sage-grouse lek location, because Coates et al. [13] found precipitation effects on lek trends were best described at a 10-km spatial resolution. To make seasonal precipitation variables, we acquired daily precipitation (mm) and snow water equivalent (kg/m^2^; SWE) from Daymet [50, 51]. We summed daily precipitation for spring (March–May), summer (June–August), and winter (December–February) and averaged SWE for spring (March–May) and winter (December–February).

In addition to spatial specificity, many of the explanatory variables had changing values across time (i.e., time-varying). We represented the temporal component of these variables as (1) stationary, (2) annually changing (e.g., precipitation, human structures), (3) annually changing but cumulative across time (e.g., fire), or (4) time-step changing (e.g., landcover variables; S1 Table). Stationary variables were not time-varying. Each time-varying variable had a single temporal component as described in S1 Table. Data source temporal reporting and collection of data dictated whether a variable was stationary or time-varying. For example, power line and road data were only reliably available as currently existing, and habitat variables could only be described with a time-step component (i.e., landcover designations from the National Land Cover Dataset [NLCD] and SPOT landcover databases were only available during a few years between 1995–2013; S1 Table). We inferred whether an annually changing variable should change annually with no addition or subtraction (harvest pressure variables and precipitation), add and subtract features annually (human population, oil and gas wells, and wind turbine density), or cumulatively aggregate (fire proportion) based on known response of sage-grouse population trends to these factors. We specified data from the Monitoring Trends in Burn Severity (MTBS) database (fire proportion) to be annually changing but cumulative with fire data starting in 1984 [52]. This was based on big sagebrush community recovery times of 25–35 years in best case scenarios and 50–120 for Wyoming big sagebrush (*A*. *t*. *wyomingensis*) communities ([53]; S1 Table).

Harvest season regulations provided a common currency for harvest exposure throughout states and provinces, which we considered indices of harvest pressure based on the relation of more liberal season regulations resulting in more harvested sage-grouse. We obtained hunting season regulations for sage-grouse from wildlife agencies in 11 western United States and two Canadian provinces (Table 2). Hunting regulation variables included annual bag and possession limits, season start dates, season lengths, number of weekends, and hunting season type (general hunting license or limited permits) from 1995–2013 (Table 2 and S1 Table). Permitted hunting seasons specified a limited number of hunters and a maximum season limit (i.e., number of birds shot). This was different than general hunting seasons with no limits on the number of hunting licenses and possession limits allow for additional days of hunting after birds are no longer in the hunter’s possession. As the legal area open to harvest was not limited to sage-grouse habitat and some sage-grouse populations were only partially exposed to legal harvest, we area-weighted bag limits, possession limits, season length, and number of weekends based on the area of each sage-grouse population to more directly relate to the maximum potential for hunting pressure (i.e., harvest exposure). We calculated the proportion of land open to harvest within 8 km of our relatively distinct sage-grouse populations. This minimized issues associated with seasonal migration of sage-grouse, which made determining differences in harvest exposure related to specific lek locations impossible. Hunting regulation variables were annual area-weighted averages within each of the 21 sage-grouse populations, because we were unable to quantify harvest exposure at smaller spatial scales (i.e., unknown harvest location and confounding of seasonal migration of sage-grouse; S1 Table).

**Table 2:** Hunting regulations stratified by regulation history and sage-grouse population. Bag/possession limits and season lengths (days) represented harvest exposure for sage-grouse. Data from western U.S. and southern Alberta and Saskatchewan Canada from 1995–2013. Footnotes highlight major changes to hunting regulations between 1995 and 2013.

Regulation history	Sage-grouse populations	1995 bag/possession	1995 season length	2013 bag/possession	2013 season length
Never_1995_	Moses Coulee, WA	0/0	0	0/0	0
	Saskatchewan, Canada	0/0	0	0/0	0
	Yakima Training Center, WA	0/0	0	0/0	0
Never_1996_	Northeast Interior, UT	0/0	0	0/0	0
	Sanpete, UT	0/0	0	0/0	0
	Sheeprock Mountain, UT	0/0	0	0/0	0
	Summit/Morgan, UT	0/0	0	0/0	0
	Weiser, ID	0/0	0	0/0	0
First-Year	Alberta, Canada	1/2	7	0/0	0
	Southwest, UT	2/4	4	0/0	0
Jackson	Jackson, WY^a^	3/6	15	0/0	0
Permit	Baker, OR	2/2	5	2/2	9
	Central, OR	2/2	5	2/2	9
	North Mono Lake, CA/NV^b^	1/1 CA	2 CA	1/1 CA	2 CA
		2/2 NV	1 NV	0/0 NV	0 NV
	Parker Mountain, UT	2/4	4	2/2	23^c^
	South Mono Lake, CA	1/1	2	1/1	2
General	Belt, MT^d^	3/12	106	2/4	62
	Middle Park, CO^e^	1/2	17	2/4	7
	North Park, CO^f^	2/4	17	2/2	2
	Southwest, MT^d^	3/12	106	2/4	62
Study-Continuous	Snake/Salmon/Beaverhead, (partial), ID^g^	3/6	30	1/2	7
Study-Discontinuous	Snake/Salmon/Beaverhead, (partial), ID^h^	3/6	30	1/2	7

^a^Bag/possession limits were 0/0, 2/4, 0/0 and season lengths were 0, 9, and 0 days for 2000–2001, 2002, 2003–2013, respectively.

^b^Nevada last hunting season 1998.

^c^Utah changed from a general upland game season to permit only hunting in 2000; thus, the exposure to harvest during the 23 day season length was minimized to 2 birds/permit.

^d^Montana reduced season length from 106 to 62 days starting in 1996. Bag/possession limits were 2/6 for 1996–1999, 3/6 for 2000–2004, 2/4 for 2005, 4/8 for 2006, and 2/4 2007–2013.

^e^Bag/possession limits and season length were 1/2 and between 16 and 22 days depending on year for 1995–1997 and 2/4 and 7 days for 1998–2013, respectively.

^f^Bag/possession limits and season length were 2/4 and between 16 and 22 days depending on year for 1995–1997, 2/4 and 7 days for 1998–2007, and 2/2 and 2 days 2008–2013, respectively.

^g^Bag/possession limit and season length were 3/6 and 30 days for 1995–2006 and 1/2 and 7 for 2007–2013, respectively.

^h^Bag/possession limits and season length were 3/6 and 30 days for 1995, 0/0 and 0 days for 1996–2001, and 1/2 and 7 days 2002–2013, respectively.

### Statistical analyses

We used open population N-mixture models created by Dail and Madsen [54] and Royle [55] to assess population change over time—quantified as lek trends while simultaneously modeling detection probability. These models were successfully used to assess sage-grouse lek trends by McCaffery et al. [56]. In addition to previous specification of population change as exponential growth (lambda [λ]), model extensions have been recently developed to allow users to incorporate underlying Gompertz or Ricker density-dependent population growth [57]. This class of models also provides inference on spatiotemporal variation in abundance based on explanatory variables, which can be specified to describe initial abundance (Λ; time = 1), population growth (instantaneous growth rate [*r*] or maximum per capita rate of increase [λ]), or equilibrium abundance (*K*; analogous to carrying capacity). Variables describing initial abundance were related to 1995 for each sampling location (e.g., lek locations) with the exception of the Never_1996_ regulation history that started in 1996; whereas, variables describing population growth or *K* were time-varying for each sampling location. Population growth rate was enumerated as λ and *r* for exponential growth and density-dependent growth, respectively [57]. Only models with underlying density-dependent growth included *K*, which was the average male count at individual leks where density-dependence effects influenced lek trend trajectory.

We conducted our analyses with the ‘pcountOpen’ function of package UNMARKED version 0.11–0 [58] in R. Model comparison was evaluated using Akaike’s information criterion (AIC) and Akaike weights (*w*_*i*_; [57, 59]). We conducted analyses separately for each regulation history to aid evaluation of potential population suppression of sage-grouse from hunter harvest by directly comparing differential population trends among regulation histories (Table 1). We first determined the best distribution (Poisson and negative binomial) and base population dynamics by comparing (1) no trend, (2) trend (exponential growth), (3) Gompertz, and (4) Ricker population growth models without explanatory variables with AIC. As observation related data (e.g., Julian date, time of day for max count, weather directly during data collection, observer experience, etc.) were lacking from most lek surveys and data available to us represented max counts from multiple repeat visits, we evaluated detection probability among years and lek locations with spatiotemporal variables quantified at coarser scales. This was an improvement from many sage-grouse lek analyses that make a common assumption that detection does not vary [3, 4, 9, 13] or simply varies by year [56]. Specifically, the best base population dynamics model had elevation, topographic ruggedness, state/province, WAFWA Management Zone (MZ), sage-grouse population, and time-varying weather variables evaluated as potential detection covariates. Weather variables as detection covariates were temporally aligned with when lek counts were conducted and enumerated as March, April, spring, or the winter directly prior to the lek count. We retained all informative detection covariates that were not correlated as ranked with AIC. There still was potential for unquantified bias in detection probability associated with small spatiotemporal processes among lek surveys (e.g., wind during a particular morning); however, lek count methodology for sage-grouse has been designed to minimize bias associated with differences in detection.

The base population dynamic model with detection covariates with the lowest AIC was carried forward to evaluate the influence of human activity (except harvest exposure) and environmental change variables on sage-grouse lek trends for each regulation history. Finally, we added each hunting regulation variable separately to the best—lowest ΔAIC—model with all other explanatory variables. Hunting regulation variables were evaluated within models parameterized with all other variables, because the potential for suppression of sage-grouse lek trends via human hunting was a central component of our study. If there was uncertainty of the best base population dynamics model without covariates, then we determined the best base model by adding precipitation variables on *r* or λ to all base models within 4 ΔAIC of the lowest AIC model. We report results of all final models within 4 ΔAIC of the model with the lowest AIC.

To reduce complexity of models with explanatory variables, we removed uninformative variables from further modeling. Explanatory variables with parameter estimates that had 85% confidence intervals (CI) that overlapped zero were considered uninformative [60]. We compared all combinations of models with informative explanatory variables on initial abundance, population growth, and *K* (i.e., *K* was only included if the best population dynamics model was density-dependent). We used empirical Bayes methods within the UNMARKED package to generate confidence intervals for lek trends [58]. We report lek trends (as number of males) by regulation history sub-stratified by WAFWA MZs. We avoided multicollinearity by examining Pearson’s correlation matrices and excluding variables with correlation coefficient > |0.65|.

We assessed the effect of human activity and environmental change variables on initial abundance (Λ) by comparing spatially explicit (relative to each lek) variables to the initial lek count year—either 1995 or 1996 (Tables 1 and 3). We included all stationary and annually changing variables (time-varying) as explanatory variables describing Λ with the exception of harvest exposure and precipitation; however, time-varying variables compared to Λ were reduced to represent a temporal alignment to 1995 or 1996 (Tables 1 and 3). Simultaneously, we evaluated the effects of time-varying hunting regulation variables and precipitation on λ or *r* and all other time-varying human activity and environmental change variables on *K* (Table 3).

**Table 3:** Sets of variables considered as predictors in models. N-mixture models included explanatory variables estimating initial abundance (Λ) were lek specific and related to 1995 (except Λ for Never_1996_ was related to 1996). Models with density-dependent dynamics (Gompertz or Ricker models) included time-varying variables predicting instantaneous growth rate (*r*) and equilibrium abundance (*K*). Whereas, models with exponential growth dynamics included time-varying variables predicting maximum per capita rate of increase (lambda [λ]). Explanatory variables were spatially explicit to each sage-grouse lek with time-varying variables aligned temporally as near to data acquisition date as possible, 1990–2013.

Initial abundance (Λ)	Instantaneous growth rate (*r*) or maximum per capita rate of increase (lambda [λ])^a^	Equilibrium abundance (*K*)^b^
Habitat^c^	Precipitation^d^	Habitat^c^
Cropland proportion	Previous year, 2-year lag	Cropland proportion
Tree proportion	Precipitation	Tree proportion
	Snow water equivalent	
Anthropogenic	Harvest regulation^e^	Anthropogenic
Human population	Harvest area	Previous year, 3-, 5-year lags
Oil and gas well	Bag/possession limits	Human population
Power line	Season length	Oil and gas wells
Road density	Season opening later than Sept 16	Wind turbines
Wind turbines		
Fire proportion		Fire proportion
		Previous year, 3-, 5-year lags
Sage-grouse Management Zone		

^a^Instantaneous growth rate (*r*) or maximum per capita rate of increase (lambda [λ]).

^b^Equilibrium abundance (*K*) is also referred to as carrying capacity.

^c^Habitat variables were quantified from NLCD and SPOT satellite landcover data. For Λ, habitat values were fixed at the earliest year possible. For *K*, variables were time-varying by lek with values changing by year for anthropogenic and fire variables and by 5 or 6 year intervals for habitat variables.

^d^Weather variables were quantified as winter (December–February), spring (March–May), and summer (June–August).

^e^Hunting regulation variables were quantified as area-weighted averages representing harvest exposure.

Time-varying variables were quantified by aligning the variable value from one year prior to a lek count. We also used annual data values from previous years to represent lag effects with a 2-year lag for precipitation and 3-year and 5-year lags for fire proportion and human population, oil and gas well, and wind turbine density (Table 3). However, time-step changing habitat variables (cropland and forested proportion) from NLCD and SPOT datasets were only available as processed snapshots (NLCD; 1992, 2001, 2006, and 2011) or combined output of 5-year timespans centered at a median year (SPOT; 1997–2002, 2003–2007, 2008–2012; S1 Table). For NLCD data, 1992 data values were temporally aligned with 1995–1998, 2001 values with 1999–2003, 2006 values with 2004–2008, and 2011 values with 2009–2013. For SPOT, 2000 date values were temporally aligned with 1995–2002, 2005 values with 2003–2007, and 2010 values with 2008–2013. For regulation histories that included male count data from Canada, we did not evaluate NLCD variables, because they were unavailable in Canada. For United States based regulation histories, we compared cropland and forested proportion variables as single variable models calculated from NLCD and SPOT datasets with AIC; the NLCD or SPOT version of cropland and forested proportion variables with the lowest AIC were used in additive modeling.

## Results

Our analysis examined lek trends for 20 small sage-grouse populations and one relatively larger sage-grouse population (Fig 1; Table 1). Lek trends spanned 1995–2013 (19 years) for all but one regulation history that included five small populations and spanned 1996–2013 (18 years; Table 1). With the exceptions of Jackson, Study-Continuous, and Study-Discontinuous, each regulation history included ≥2 MZ and ≥2 sage-grouse populations (Fig 1; Table 1). The Study-Continuous and Study-Discontinuous regulation histories were encompassed within the same relatively larger sage-grouse population and each represented approximately one third of the spatial footprint of the Snake/Salmon/Beaverhead sage-grouse population (Fig 1). However, both of these regulation histories—each representing a partial sage-grouse population—were relatively separated from each other and the remaining third of the Snake/Salmon/Beaverhead population by areas of low lek density and less suitable habitat (i.e., less shrub cover and greater topographical ruggedness). All other regulation histories included the entirety of ≥1 smaller sage-grouse population (Fig 1; Table 1). A total 935 leks were provided in the WAFWA and wildlife agency (state and provincial) datasets, which we reduced to 400 (43%) based on our lek data restrictions. The sample of leks within each regulation history was ≥25 with the exception of the Jackson regulation history with 8 leks (Table 1).

### Base population dynamics

There was no support for models lacking exponential growth or density-dependence for any of the regulation histories (i.e., no trend models were at least ΔAIC>411 compared to density-dependent base models). Time-varying weather variables from the winter prior to (Never_1995_, Never_1996_, First-Year, General) or spring when leks were being counted (Jackson, Permit, Study-Continuous, Study-Discontinuous) described detection probability with negative and positive relationship, which likely relate to 1) observers ability to access lek locations and see all birds or 2) bird behavior. Precipitation was positively related to detection in the Never_1996_ (winter), Permit (spring), Study-Continuous harvest histories (spring), but negatively related in the General (Colorado and southwestern Montana) harvest history. Snow water equivalent was negatively related to detection in Never_1995_ (winter), Jackson (spring), and Study-Discontinuous (spring) harvest histories; however, it was positively related to detection in the First-Year (winter) harvest history. Winter and spring precipitation represented generalizations of detection probability for each year. The single best fitting precipitation or SWE variable was included in all other additive models. We used the negative binomial rather than the Poisson distribution for all models based on consistently lower AIC values. Density-dependent models generally fit data better as base models than exponential growth. The Never_1995_ was the only regulation history with a density-dependent base model where Ricker fit the data better than Gompertz (ΔAIC = 2.70).

For the First-Year and Jackson regulation histories, we included precipitation variables and then compared trend (exponential growth) and Gompertz models to clarify the best base model form. The First-Year regulation history had uncertainty with the best base model of exponential growth or Gompertz density-dependence (ΔAIC ≤2). However, Gompertz growth fit the First-Year regulation history data better than exponential growth (ΔAIC = 14.96) after inclusion of precipitation variables on *r* or λ. The Jackson sage-grouse population also had high uncertainty with the best base model (ΔAIC ≤2), and it was the only regulation history where trend fit the data better than a density-dependent base model (ΔAIC = 17.44) after inclusion of precipitation variables describing *r* or λ (Table 4). Our Jackson regulation history results may be indicative of no major habitat differences or changes among leks influencing *K*, 1995–2013, or too much variability for the small sample of leks. However, there was little spatiotemporal variability in habitat-based environmental change (proportion forested or burned habitat) or human activity variables in the Jackson sage-grouse population.

**Table 4:** Rankings of N-mixture models for each regulation history. Rankings were stratified into model sets comparing 1) lek-specific variables describing lambda (λ) or instantaneous growth rate (*r*) and equilibrium abundance (*K*; top five models reported), and 2) top-lek specific variable model with addition of population specific variables (hunting regulation) on *r* or λ (top five models reported). Base population dynamics of no trend, trend (exponential growth), and density-dependence (Gompertz [Gomp] and Ricker [Rick]), weather effects on *r*, and detection covariates were selected prior to comparison of lek and population specific variables. Male count data were collected throughout the western U.S. and southern Alberta and Saskatchewan, Canada from 1995–2013.

Models^a^	*K*	AIC	ΔAIC	*w*_*i*_
**Never**_**1995**_ **: Λ, *r*, and *K*** ^b^ ^**,**^ ^c^				
Λ(MZ)+Rick[*r*(SWE_winL2_+PREC_sumL2_)+*K*(HUM)]+*p*(SWE_win_)	10	3,106.7	0.00	0.46
Λ(MZ)+Rick[*r*(SWE_winL2_+PREC_sumL2_)+*K*(FIRE)]+*p*(SWE_win_)	10	3,108.1	1.34	0.24
Λ(MZ)+Rick[*r*(SWE_winL2_+PREC_sumL2_)+*K*(HUM+FIRE)]+ *p*(SWE_win_)	11	3,108.4	1.71	0.20
Λ(MZ)+Rick[*r*(SWE_winL2_+PREC_sumL2_)+*K*(.)]+*p*(SWE_win_)	9	3,109.6	2.92	0.11
Λ(.)+Rick[*r*(SWE_winL2_+PREC_sumL2_)+*K*(.)]+*p*(SWE_win_)	8	3,117.7	10.99	0.00
**Never**_**1996**_ **: Λ, *r*, and *K*** ^c^				
Λ(MZ)+Gomp[*r*(PREC_spr2_)+*K*(sCROP+OIL_L5_+FIRE_L5_)]+ *p*(PREC_win_)	11	4,896.8	0.00	0.84
Λ(MZ)+Gomp[*r*(PREC_sprL2_)+*K*(sCROP+FIRE_L5_)]+*p*(PREC_win_)	10	4,900.4	3.53	0.14
Λ(MZ)+Gomp[*r*(PREC_sprL2_)+*K*(OIL_L5_+FIRE_L5_)]+*p*(PREC_win_)	10	4,905.9	9.05	0.01
Λ(MZ)+Gomp[*r*(PREC_sprL2_)+*K*(FIRE_L5_)]+*p*(PREC_win_)	9	4,908.1	11.24	0.00
Λ(MZ)+Gomp[*r*(PREC_spr2_)+*K*(sCROP)]+*p*(PREC_win_)	9	4,917.7	20.89	0.00
**First-Year: Λ, *r*, and *K*** ^b^ ^**,**^ ^c^ ^**,**^ ^d^				
Λ(MZ)+Gomp[*r*(SWE_winL2_)+*K*(OIL_L5_+FIRE_L5_)]+*p*(SWE_win_)	10	2,768.9	0.00	0.50
Λ(MZ)+Gomp[*r*(SWE_winL2_)+*K*(FIRE_L5_)]+*p*(SWE_win_)	9	2,769.9	1.05	0.30
Λ(MZ)+Gomp[*r*(SWE_winL2_)+*K*(OIL_L5_)]+*p*(SWE_win_)	9	2,771.6	2.70	0.13
Λ(MZ)+Gomp[*r*(SWE_winL2_)+*K*(.)]+*p*(SWE_win_)	8	2,772.7	3.77	0.08
Λ(MZ)+Gomp[*r*(.)+*K*(.)]+*p*(SWE_win_)	7	2,804.6	35.68	0.00
**Jackson: Λ and λ**				
Λ(.)+Trend[λ(PREC_sprL1_)]+*p*(SWE_spr_)	6	1,022.23	0.00	1.00
Λ(.)+Trend[λ(PREC_sprL1_)]+*p*(SWE_spr_)	5	1,039.67	17.44	0.00
Λ(.)+Trend[λ(PREC_sprL1_)]+*p*(SWE_spr_)	4	1,043.84	21.61	0.00
Λ(.)+Trend[λ(PREC_sprL1_)]+*p*(SWE_spr_)	5	1,048.18	25.95	0.00
Λ(.)+Trend[λ(PREC_sprL1_)]+*p*(SWE_spr_)	5	1,048.21	25.98	0.00
**Jackson: Λ and λ top AIC with harvest variables** ^c^				
Λ(.)+Trend[λ(PREC_sprL1_)]+*p*(SWE_spr_)	6	1,022.23	0.00	0.76
Λ(.)+Trend[λ(PREC_sprL1_+ SLEN)]+*p*(SWE_spr_)	7	1,026.03	3.79	0.11
Λ(.)+Trend[λ(PREC_sprL1_+BAG)]+*p*(SWE_spr_)	7	1,026.76	4.53	0.08
Λ(.)+Trend[λ(PREC_sprL1_+POSS)]+*p*(SWE_spr_)	7	1,027.68	5.45	0.05
Λ(.)+Trend[λ(PREC_sprL1_+HAREA)]+*p*(SWE_spr_)	5	1,039.67	17.44	0.00
**Permit: Λ, *r*, and *K*** ^c^				
Λ(.)+Gomp[*r*(SWE_winL1_)+*K*(sCROP+HUM+FIRE_L3_)]+*p*(PREC_spr_+POP)	14	16,715.5	0.00	0.93
Λ(.)+Gomp[*r*(SWE_winL1_)+*K*(sCROP+HUM)]+*p*(PREC_spr_+POP)	13	16,721.6	6.18	0.04
Λ(.)+Gomp[*r*(SWE_winL1_)+*K*(HUM+FIRE_L3_)]+*p*(PREC_spr_+POP)	13	16,723.9	8.43	0.01
Λ(.)+Gomp[*r*(SWE_winL1_)+*K*(sCROP+FIRE_L3_)]+*p*(PREC_spr_+POP)	13	16,724.0	8.58	0.01
Λ(.)+Gomp[*r*(SWE_winL1_)+*K*(sCROP)]+*p*(PREC_spr_+POP)	12	16,726.8	11.34	0.00
**Permit: Λ, *r*, and *K* top AIC with harvest variables**				
Λ(.)+Gomp[*r*(SWE_winL1_+PERMIT)+*K*(HUM+FIRE_L3_)]+ *p*(PREC_spr_+POP)	14	16,674.0	0.00	1.00
Λ(.)+Gomp[*r*(SWE_winL1_+SLEN)+*K*(HUM+FIRE_L3_)]+*p*(PREC_spr_+ POP)	14	16,698.9	24.86	0.00
Λ(.)+Gomp[*r*(SWE_winL1_+BAG)+*K*(HUM+FIRE_L3_)]+*p*(PREC_spr_+ POP)	14	16,710.5	36.51	0.00
Λ(.)+Gomp[*r*(SWE_winL1_)+*K*(sCROP+HUM+FIRE_L3_)]+*p*(PREC_spr_+ POP)	14	16,715.5	41.44	0.00
Λ(.)+Gomp[*r*(SWE_winL1_+POSS)+*K*(sCROP+HUM+FIRE_L3_)]+*p*(PREC_spr_+POP)	14	16,723.9	49.85	0.00
**General: Λ, *r*, and *K*** ^c^				
Λ(nTREE)+Gomp[*r*(PREC_sprL2_)+*K*(nTREE)]+*p*(PREC_win_+POP)	12	11,913.6	0.00	0.74
Λ(.)+Gomp[*r*(PREC_sprL2_)+*K*(nTREE)]+*p*(PREC_win_+POP)	11	11,915.7	2.06	0.26
Λ(nTREE)+Gomp[*r*(PREC_sprL2_)+*K*(.)]+*p*(PREC_win_+POP)	11	11,928.1	14.48	0.00
Λ(.)+Gomp[*r*(PREC_sprL2_)+*K*(.)]+*p*(PREC_win_+POP)	10	11,930.0	16.43	0.00
Λ(.)+Gomp[*r*(.)+*K*(.)]+*p*(PREC_win_+POP)	9	11,942.5	28.96	0.00
**General: Λ, *r*, and *K* top AIC with harvest variables**				
Λ(nTREE)+Gomp[*r*(PREC_sprL2_+POSS)+*K*(nTREE)]+*p*(PREC_win_+ POP)	13	11,906.1	0.00	0.94
Λ(nTREE)+Gomp[*r*(PREC_sprL2_)+*K*(nTREE)]+*p*(PREC_win_+POP)	12	11,913.6	7.53	0.02
Λ(nTREE)+Gomp[*r*(PREC_sprL2_+SLEN)+*K*(nTREE)]+(PREC_win_+ POP)	13	11,914.4	8.33	0.01
Λ(nTREE)+Gomp[*r*(PREC_sprL2_+BAG)+*K*(nTREE)]+*p*(PREC_win_+ POP)	13	11,914.7	8.64	0.01
Λ(nTREE)+Gomp[*r*(PREC_sprL2_+HAREA)+*K*(nTREE)]+ *p*(PREC_win_+POP)	13	11,915.7	9.68	0.01
**Study-Continuous: Λ, *r*, and *K*** ^c^				
Λ(.)+Gomp[*r*(PREC_sprL1_)+*K*(sCROP+FIRE_L5_)]+*p*(SWE_spr_)	9	8,571.3	0.00	0.48
Λ(.)+Gomp[*r*(PREC_sprL1_)+*K*(sCROP+HUM+FIRE_L5_)]+*p*(SWE_spr_)	10	8,571.8	0.51	0.52
Λ(.)+Gomp[*r*(PREC_sprL1_)+*K*(sCROP+HUM)]+*p*(SWE_spr_)	9	8,597.58	26.26	0.00
Λ(.)+Gomp[*r*(PREC_sprL1_)+*K*(HUM+FIRE_L5_)]+*p*(SWE_spr_)	9	8,608.3	36.98	0.00
Λ(.)+Gomp[*r*(PREC_sprL1_)+*K*(.)]+*p*(SWE_spr_)	7	8,634.37	63.05	0.00
**Study-Continuous: Λ, *r*, and *K* top AIC with harvest variables**				
Λ(.)+Gomp[*r*(PREC_sprL1_)+*K*(sCROP+FIRE_L5_)]+*p*(SWE_spr_)	9	8,571.3	0.00	0.52
Λ(.)+Gomp[*r*(PREC_sprL1_+BAG)+*K*(sCROP+FIRE_L5_)]+*p*(SWE_spr_)	10	8,573.6	2.31	0.16
Λ(.)+Gomp[*r*(PREC_sprL1_+SLEN)+*K*(sCROP+FIRE_L5_)]+*p*(SWE_spr_)	10	8,573.7	2.35	0.16
Λ(.)+Gomp[*r*(PREC_sprL1_+POSS)+*K*(sCROP+FIRE_L5_)]+*p*(SWE_spr_)	10	8,573.7	2.38	0.16
Λ(.)+Gomp[*r*(PREC_sprL1_)+*K*(.)]+*p*(SWE_spr_)	7	8,634.37	63.05	0.00
**Study-Discontinuous: Λ, *r*, and *K*** ^c^				
Λ(.)+Gomp[*r*(SWE_winL2_)+*K*(sCROP)]+*p*(PREC_win_)	8	5,657.0	0.00	0.76
Λ(.)+Gomp[*r*(SWE_winL2_)+*K*(.)]+*p*(PREC_win_)	7	5,659.4	2.34	0.23
Λ(.)+Gomp[*r*(SWE_winL2_)+*K*(.)]+*p*(.)	6	5,665.7	8.64	0.01
Λ(.)+Gomp[*r*(.)+*K*(.)]+*p*(PREC_win_)	6	5,680.5	23.47	0.00
Λ(.)+Gomp[*r*(.)+*K*(.)]+*p*(.)	5	5,703.2	46.30	0.00
**Study-Discontinuous: Λ, *r*, and *K* top AIC with harvest variables**				
Λ(.)+Gomp[*r*(SWE_winL2_+SLEN)+*K*(.)]+*p*(PREC_win_)	8	5,637.2	0.00	0.34
Λ(.)+Gomp[*r*(SWE_winL2_+BAG)+*K*(.)]+*p*(PREC_win_)	8	5,637.3	0.10	0.33
Λ(.)+Gomp[*r*(SWE_winL2_+POSS)+*K*(.)]+*p*(PREC_win_)	8	5,637.3	0.10	0.33
Λ(.)+Gomp[*r*(SWE_winL2_+SLEN)+*K*(sCROP)]+*p*(PREC_win_)	9	5,639.0	1.86	0.14
Λ(.)+Gomp[*r*(SWE_winL2_+BAG)+*K*(sCROP)]+*p*(PREC_win_)	9	5,639.5	1.94	0.13

^a^Models without population dynamics were not competitive (>410 ΔAIC).

^b^Only SPOT habitat variables were assessed, because NLCD does not extend into southern Canada.

^c^Few covariates on Λ were informative; thus, we only report models with the best AIC ranked Λ variables. Only the best single or additive version of λ or *r* weather covariates were reported. Lag effects for time-varying variables are denoted as 2-year lag (_L2_), 3-year lag (_L3_), and 5-year lag (_L5_).

^d^Top AIC Gompertz model with SWE_winL2_ on *r* was ΔAIC = 14.96 lower than best AIC trend model with PREC_sumL2_ on λ; thus, modeling of *r* and *K* was done with Gompertz dynamics.

### Environmental change and human activities

Models with either environmental change and/or human activity variables fit each regulation history better than base population dynamic models alone (Table 4). Either environmental change and/or human activity variables describing λ or *r* and *K* more commonly fit lek trend data from all regulation histories compared to models including environmental change and human activity variables describing Λ (Table 4). This indicated that annually changing or time-step changing variables better described lek trends across time compared to differences in initial male counts quantified with stationary variables. For cropland and forested landcover proportion variables (Λ or *K*), directionality and magnitude of parameter estimates from single variable models were similar regardless of quantification with NLCD or SPOT datasets, which indicated both datasets quantified change in cropland and forested habitat similarly in regulation histories where both datasets were available (i.e., sage-grouse populations in the United States). Our top AIC selected models with covariates describing Λ, λ or *r*, and *K* did not have high multicollinearity as no pair of variables had a correlation coefficient >|0.65|. Parameter estimate directionality was consistent, and magnitude remained relatively stable when comparing additive models to single variable models.

**Fig 2:**
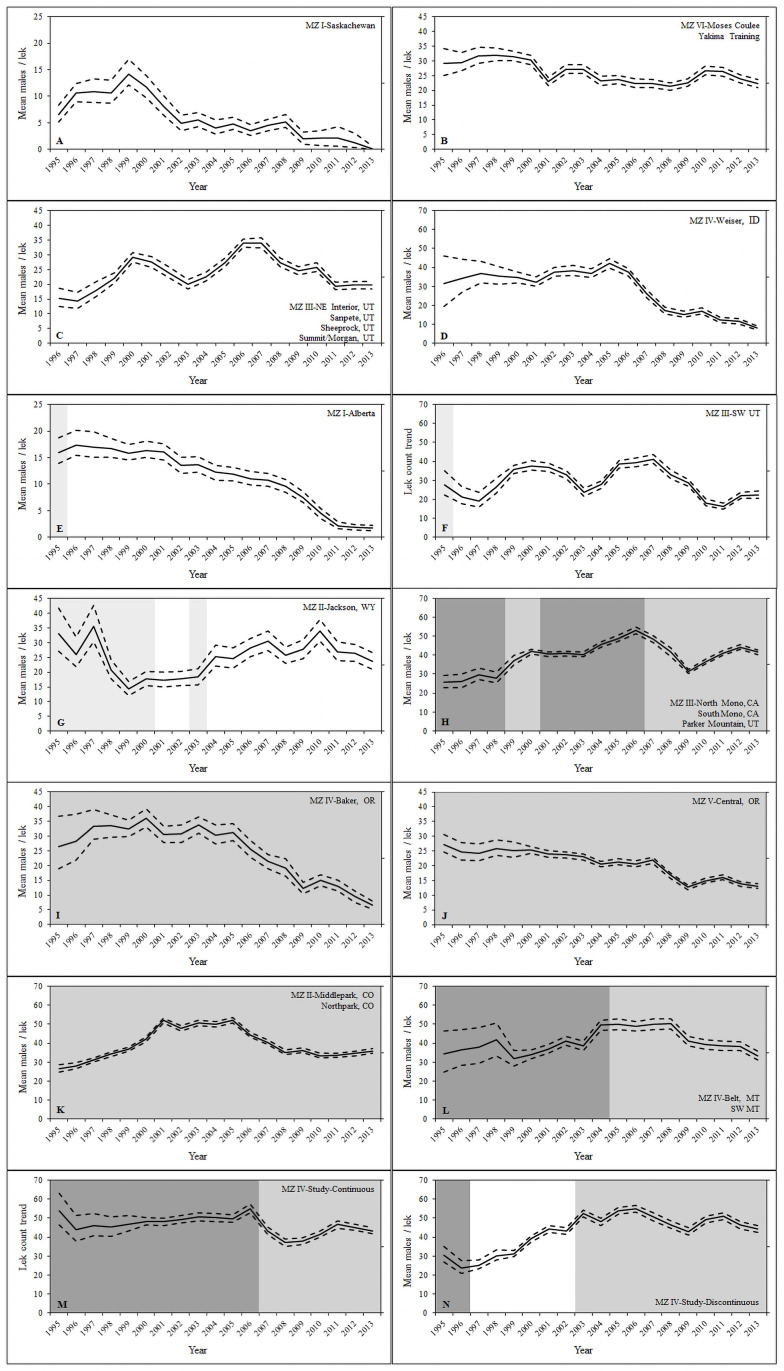
Predicted lek counts (mean males/lek) during 1995–2013 from top open population N-mixture models using empirical Bayes methods. Annual predictions were stratified by regulation history and sage-grouse Management Zone (MZ). Light gray indicates human harvest occurred but no harvest exposure effect was detected, medium gray indicates that a harvest exposure variable was influential on instantaneous growth rate (*r*) with harvest exposure relatively low, and dark gray indicates a harvest exposure variable was influential on instantaneous growth rate (*r*) with harvest exposure relatively high. Lek count trend predictions were included for Never_1995_ (A) and (B), Never_1996_ (C) and (D), First-Year (E) and (F), Jackson (G), Permit (H), (I), and (J), Upland (K) and (L), Study-Continuous (M), and Study-Discontinuous (N). Male count data were collected by states and provinces throughout the western United States and southern Alberta and Saskatchewan Canada from 1995–2013.

**Fig 3:**
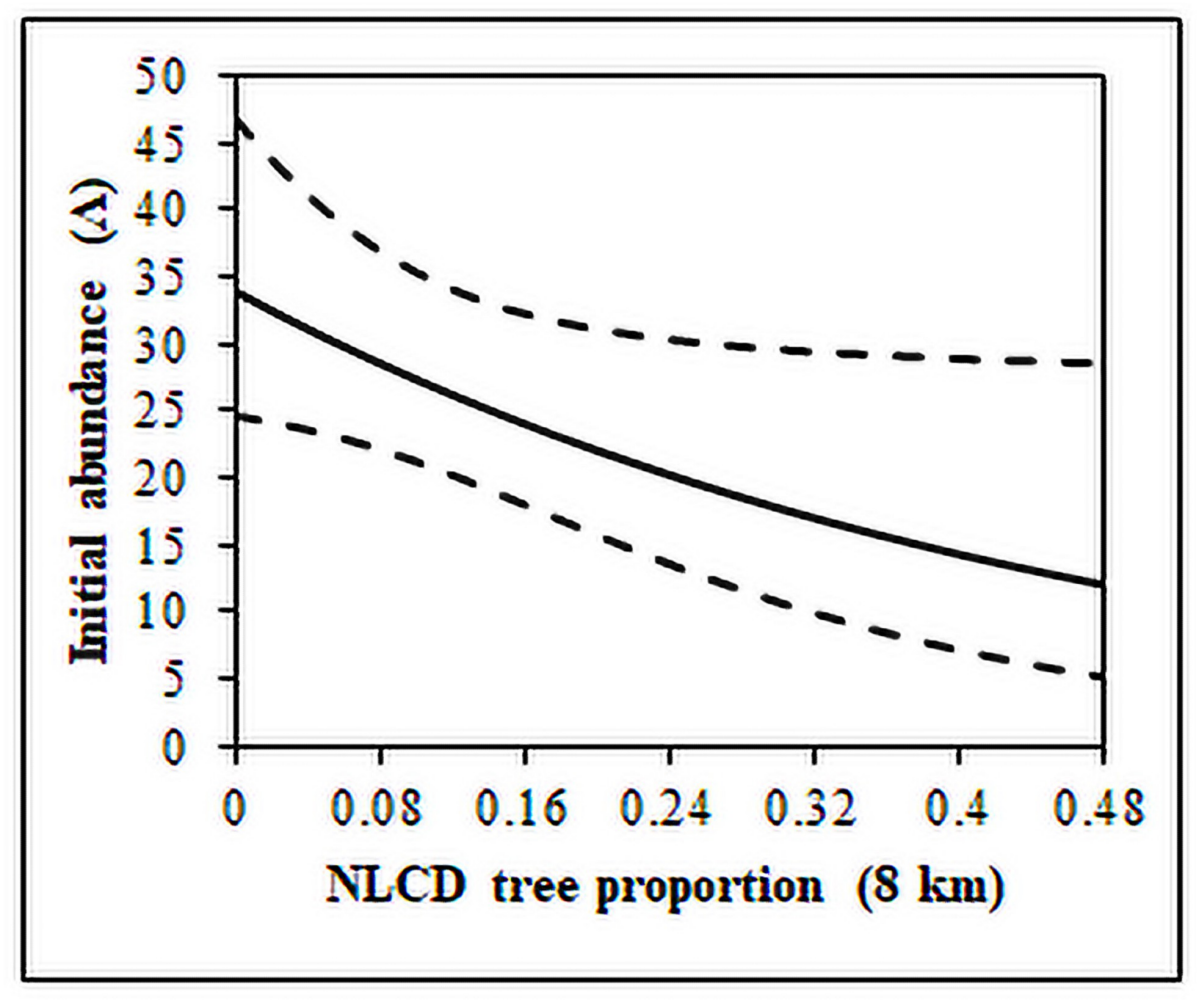
Predicted effect of tree proportion (NLCD) within 8 km of a sage-grouse lek on initial abundance (Λ) within the Upland regulation history. Prediction was from top ΔAIC open population N-mixture model for the Upland regulation history with Λ lek specific and related to 1995.

Our results indicated that Λ was generally similar for leks within each regulation history during the initial year (1995 or 1996) with differences among MZ for Never_1995_, Never_1996_, and First-Year regulation histories (Table 4). These slight differences by MZ were on average 3.19 (SE = 1.38) males per lek more for MZ VI versus MZ I, on average 2.87 (SE = 1.37) males per lek more for MZ IV versus MZ III, and on average 1.60 (SE = 1.37) males per lek more for MZ III versus MZ I for the Never_1995_, Never_1996_, and First-Year regulation histories, respectively. Permit and General regulation histories also had >1 MZ represented; however, we did not detect differences in average initial lek count for these regulation histories (Table 4). Thus, Λ at leks within Permit and General regulation histories was similar relative to our environmental change and human activity variables, or there was a greater degree of uncertainty about the estimated average initial lek counts (i.e., there may have been too many missing male count data from the initial year to estimate the effects of environmental change and human activity variables on Λ for these sage-grouse populations; Fig 2H–2K). We did not find evidence of stationary variables (distance to a town, distance to roads, and power line density) influencing Λ, which indicated Λ at leks within most regulation histories was not different or variability and missing lek data from 1995 or 1996 disallowed us from detecting differences in Λ based on stationary environmental change and/or human activity variables (Fig 2 and Table 4). However, Λ of leks within the General regulation history was better described by proportion of forested habitat (based on the initial value of time-step changing proportion of forested habitat) rather than differences in MZ (ΔAIC = 2.80), which indicated that leks with greater proportion of forested habitat within 8 km had initially lower lek counts (Fig 3). In addition to describing differences in Λ for all leks within the General regulation history, the negative effect of the proportion of forested habitat on Λ was likely describing some of the difference between Λ in MZ I and MZ II, because MZ I had lower initial lek counts and higher proportion forested habitat (average = 0.11 [0.02 SE]) compared to MZ II with higher lek counts and lower proportion forested habitat (average = 0.06 [0.01 SE]).

**Fig 4:**
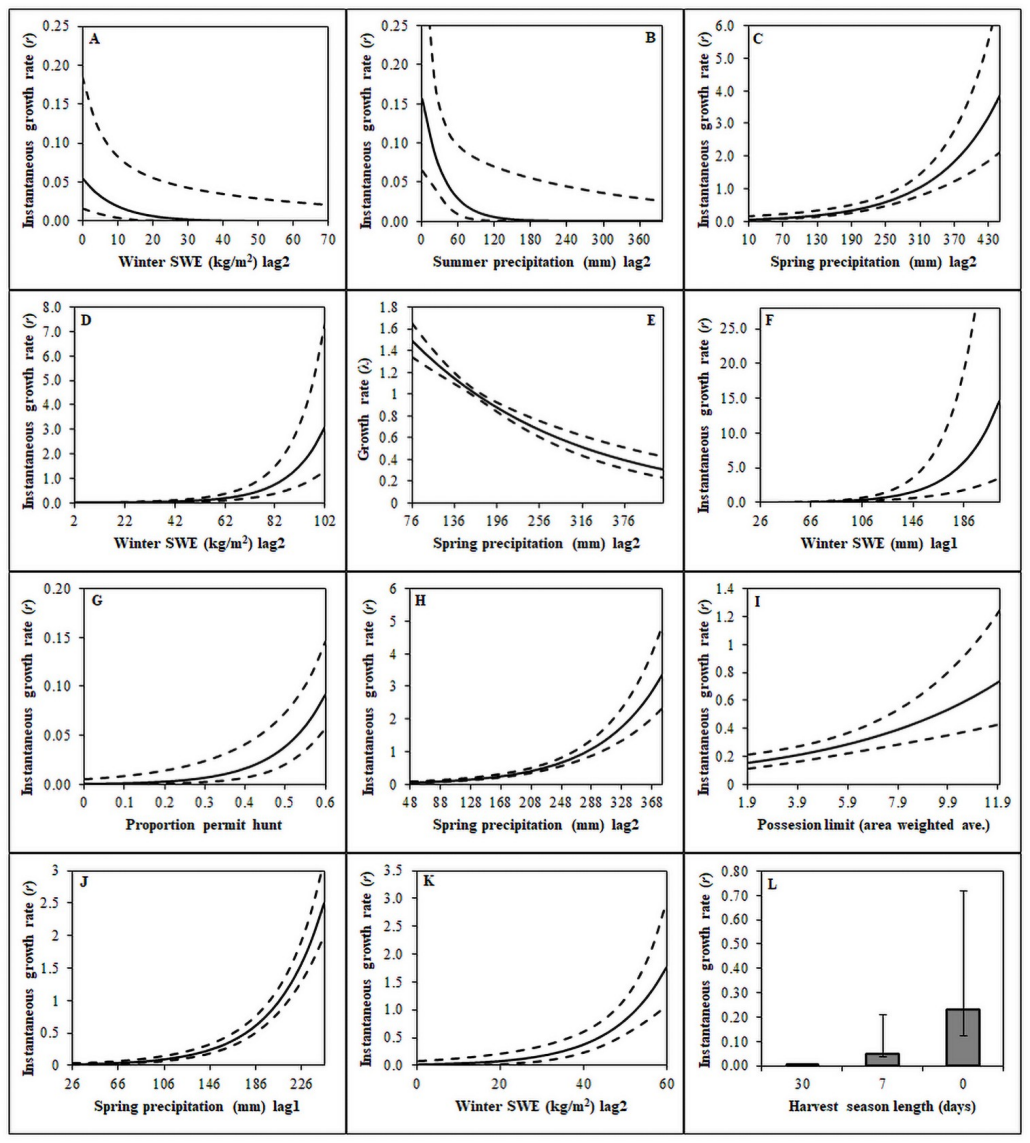
Predicted effects of weather and harvest exposure variables on the instantaneous growth rate (*r*) or maximum per capita rate of increase (lambda [λ]) from open population N-mixture models. Variables are labeled on the x-axis and refer to the following regulation histories: Never_1995_ (A) and (B), Never_1996_ (C), First-Year (D), Jackson (E), Permit (F) and (G), Upland (H) and (I), Study-Continuous (J), and Study-Discontinuous (K) and (L). Male count data were collected by states and provinces throughout the western United States and southern Alberta and Saskatchewan Canada from 1995–2013.

All of our competitive models for each regulation history included at least one precipitation variable describing λ or *r*—the top AIC selected model with Never_1995_ having two precipitation variables (Table 4). Precipitation variables were positively associated with population growth for the Never_1996_, First-Year, Permit, General, Study-Continuous, and Study-Discontinuous regulation histories (Fig 4). However, SWE in winter (SWE_winterL2_) and summer precipitation (PREC_summerL2_) with 2-year lag effects were negatively associated with *r* for Never_1995_, and spring precipitation (PREC_springL1_) with 1-year lag effects were negatively related to λ in Jackson (Fig 4). No regulation history included both 1-year and 2-year lag effects on different precipitation variables in the same model (Table 4). Three regulation histories had precipitation variables with 1-year lag effects, and five regulation histories had precipitation variables with 2-year lag effects (Table 4). However, these lag effects were not related to the best fitting lag timeframe for environmental change and/or human activity variables, because regulation histories with 1-year lag effects on precipitation had either shorter or longer lag effects on variables influencing *K*, which was also true for regulation histories with 2-year lag effects on precipitation (Table 4).

**Fig 5:**
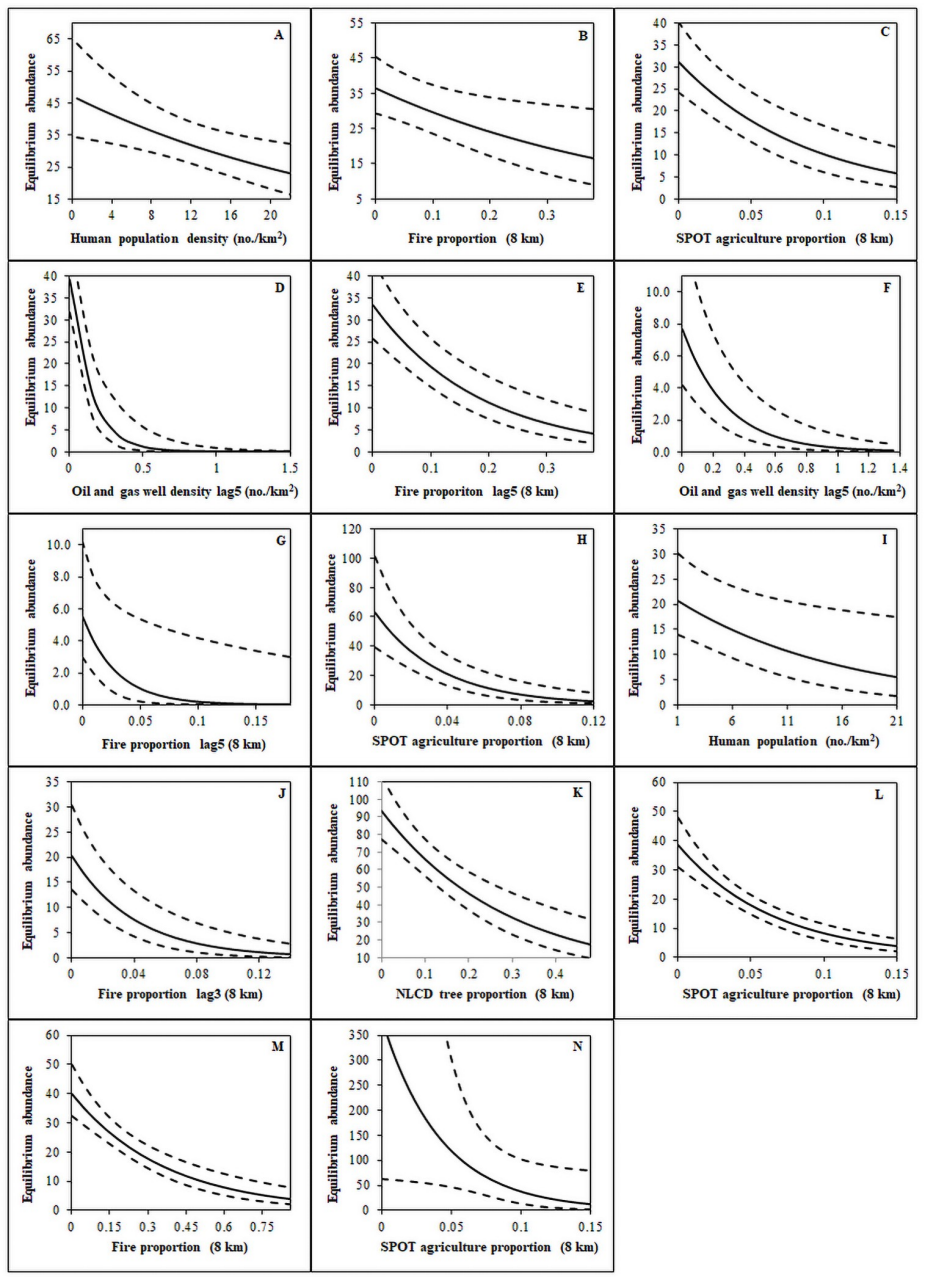
Predicted effects of anthropogenic, fire, and habitat variables on equilibrium abundance (*K*) from open population N-mixture models (Gompertz or Ricker density-dependent models). Variables are labeled on the x-axis and refer to the following regulation histories: Never_1995_ ((A) top ΔAIC model and (B) second ΔAIC model); Never_1996_ (C), (D), and (E); First-Year (F) and (G); Permit (H), (I), and (J); Upland (K); Study-Continuous (L) and (M); and Study-Discontinuous (N). Male count data were collected by states and provinces throughout the western United States and southern Alberta and Saskatchewan Canada from 1995–2013.

Most of our top AIC selected models for each regulation history included environmental change and human activity variables with the exception of the Jackson regulation history, which only included variables describing environmental change (Table 4). The cropland variable was informative and negatively related to *K* for the Permit and Study-Discontinuous harvest histories (Fig 4); however, the cropland variable could not be included in the top model with harvest variables as the beta parameter had signs of multicollinearity with sign change or parameter precision vastly reducing. We report on the two lowest ΔAIC (ΔAIC ≤ 1.34) models for the Never_1995_ regulation history, because those models included different annually changing variables describing *K* (human population density and fire proportion from the previous year), and there was high uncertainty about the model best describing the data (Table 4; Fig 5). We report model inference from the model with the lowest ΔAIC for all other regulation histories (Table 4). Habitat-based environmental change variables were in most competitive models describing *K*; however, the Jackson and Study-Discontinuous regulation histories did not have a habitat-based environmental change variable describing *K* (Table 4). Our models indicated that higher proportions of burned (Never_1995_, Never_1996_, First-Year, Permit, and Study-Continuous regulation histories) and forested (General regulation history) habitat were associated with lower equilibrium abundance (Fig 5). Human activity variables related to sagebrush habitat loss were negatively associated with *K*, including higher proportion of cropland (Never_1996_, Permit, Study-Continuous, and Study-Discontinuous regulation histories) and oil and gas well densities (Never_1996_ and First-Year as 5-year lag effects; Fig 5). Whereas, human population density was negatively associated with *K* in the Never_1995_ and Permit regulation histories.

### Hunting regulations

Non-hunted regulation histories (*n* = 2) consisted of sage-grouse populations that had complete hunting season closures after fall of 1990 or earlier (Table 1). The three regulation histories with discontinued hunting seasons were completely terminated after fall 1995 for the First-Year, closed fall 2000 and fall 2001 then permanently closed after fall 2002 for Jackson, and closed to hunting fall 1996 through fall 2001 for Study-Discontinuous (Table 1). Continuously hunted sage-grouse populations within the Permit, General, and Study-Continuous regulation histories were exposed to harvest during all years of our study. However, state and provincial wildlife agencies restricted hunting regulations in all 13 of the relatively distinct sage-grouse populations that were exposed to hunting, 1995–2013 (Table 1). The level of harvest exposure reduction—as quantified by hunting regulation changes—was variable with complete closure of hunting seasons in the First-Year, Jackson, and Nevada portion of the Permit regulation histories compared to relatively small reductions in harvest exposure in sage-grouse populations regulated by permit only hunting in Oregon (Permit regulation history; Table 1).

We evaluated whether hunting resulted in population suppression by comparing predicted lek trends from top AIC-ranked models stratified by regulation histories and MZs relative to years of hunting closure (Fig 2; Table 4). Patterns from our assessment of differential lek trends by regulation history were not conclusive or consistent with regard to the effect of hunting exposure (Fig 2). Never_1995_ and Never_1996_ regulation histories were closed to hunting for ≥8 years before fall 1995, and MZs I, IV, and VI within these regulation histories had overall gradually to drastically decreasing lek trends, 1995–2013 (Fig 2A, 2B and 2D). Comparatively, MZ III for Never_1996_ had an overall lek trend increase with relatively more oscillation in lek trend across time (Fig 2C). There was no consistent indication of population suppression from hunting in the First-Year regulation history, because the Alberta, Canada population had an increasing average lek count, and the southwest Utah population had a decreasing average lek count following the last hunting season (Fig 2E and 2F). The lek trend from the Jackson regulation history in MZ II was decreasing from 1995–1999 then increased from 1999–2001 with harvest seasons occurring the previous fall (Fig 2G); there was continued lek trend increase from 2001–2006 while hunting was closed for fall 2000 and fall 2001, reopened harvest season in fall 2002, then permanent harvest season closure thereafter (Fig 2G). We found complete closure of hunting, 1996–2001, in the Study-Discontinuous regulation history resulted in increasing lek trends during 1997–2006 with the lek trend stabilizing a few years after the hunting season was reopened in fall 2002 (Fig 2N). This was in direct comparison to the Study-Continuous regulation history, which had a relatively stable lek trend during 1995–2006 (Fig 2M). Both the Study-Continuous and Study-Discontinuous regulation histories had nearly identical lek trends during 2007–2013 while relative harvest exposure was lower (i.e., season lengths of 7 days compared to 30 days in previous years). Lek trends increased in MZ III of the Permit regulation history, which was likely connected with complete season closure for the Nevada portion of the North and South Mono sage-grouse populations following fall 1998 and Parker Mountain sage-grouse population changing from a general hunt to permit only starting in fall 2000 (Fig 2H). In contrast, other MZs (IV and V) within the Permit regulation history had decreasing lek trends, 1995–2013 (Fig 2I and 2J). Even though state and provincial wildlife management agencies reduced the potential harvest exposure by reducing bag/possession limits, season lengths or permit numbers, both MZ II and IV of the General regulation history had oscillating lek trends with no overall pattern of increase or decrease (Fig 2K and 2L).

Harvest exposure variables were enumerated for regulation histories where hunting seasons occurred between 1995 and 2013. However, we did not evaluate area-weighted average harvest exposure variables on *r* for the Never_1995_, Never_1996_, or First-Year regulation history. By definition, our regulation histories without hunting seasons inherently had consistent non-influence of hunting across our study timeframe, 1995–2013 (Table 1). The First-Year regulation history was only subjected to hunting during fall 1995. Thus, any version of a harvest exposure variable would primarily compare growth between 1995 and 1996 and all other years (i.e., there was not a long enough record or spatiotemporal variability in annual harvest exposure to meaningfully assess the effects of harvest exposure). We found mixed results regarding the effect of potential harvest exposure on λ or *r* from the Jackson, Permit, General, Study-Continuous, and Study-Discontinuous regulation histories. For the Jackson regulation history, our model with the lowest AIC did not include a harvest exposure variable (Table 4). The proportion of a sage-grouse population open to permit only hunting (PERMIT) was positively associated with *r* for the Permit regulation history, and the model including PERMIT fit the data better than the best AIC-ranked model without a harvest exposure variable (ΔAIC = 32.10; Fig 4G and Table 4). Area-weighted possession limit (POSS) was positively associated with *r* for the General regulation history, and the model with POSS fit the data better than the best AIC-ranked model without a harvest exposure variable (ΔAIC = 7.53; Fig 4I and Table 4). The cessation of harvest from 1996–2001 in the Study-Discontinuous regulation history was associated with higher *r*. This result indicated that after accounting for other environmental change and human activity factors years with 7-day and 30-day season lengths had increasingly lower *r* (Fig 4L).

## Discussion

We assessed effects of multiple environmental change and human activity variables on initial abundance, population growth (λ or *r*), and *K* of sage-grouse populations. Effects were quantified at individual leks with the exception of harvest exposure, which was evaluated among years and populations. Our analyses provided similar results to previous studies with lek counts negatively related to environmental change and human activities that resulted in sagebrush habitat loss. Higher proportions of burnt, forested, and cropland; and greater oil and gas well densities were associated with decreasing equilibrium abundance (Fig 5). Precipitation during the spring, summer, and winter were assessed to describe oscillations in lek trends over time, which were positively associated with annual population growth with a few exceptions (Fig 4). The addition of hunting as another human activity with potential to influence population growth was unique to our lek analyses. Small populations were advantageous for our analyses, because all large populations were continuously hunted except the Snake/Salmon/Beaverhead population (Study-Discontinuous and Study-Continuous regulation histories). Effects of human harvest on lek trends were not consistent among our regulation histories (Figs 2 and 4). However, these patterns were based on small sage-grouse populations that did not have a dedicated experimental design intended to study human harvest. However, hunting in the Study-Discontinuous regulation history was negatively related to *r* compared to the Study-Continuous regulation history.

We initiated our study with the goal of evaluating multiple factors influencing sage-grouse populations with a novel inclusion of fall hunter harvest on sage-grouse. Some issues relevant to including hunting included fall populations were different than spring populations, and harvest take statistics reported in the 1970s and 1980s were inflated and thus unreliable [33]. Sage-grouse often exhibit inter-seasonal migration away from leks where they were captured [41, 61], which does not allow the effects of hunter harvest to be assigned to individual leks. To account for these issues, our analyses were designed to compare different sage-grouse population trends and relate those trends to variation in harvest exposure, which is how management agencies influence harvest pressure (Table 2). Higher growth rates during years with relatively more liberal hunting regulations could be interpreted as states liberalizing hunting regulations during periods of higher sage-grouse growth rates, which would confound interpretations of harvest effects. However, management agencies suggested this pattern was unlikely or coincidental as they did not manipulate sage-grouse hunting seasons in response to annual population size in the populations within our analyses, rather changes in hunting seasons occurred over larger timeframes with the goal of reducing exposure to be overall more conservative with harvest. We capitalized on assessing lek trends and harvest exposure in a population that included a study designed specifically to study hunting effects (i.e., Study-Discontinuous and Study-Continuous; original study conducted by Connelly et al. [30]).

The cessation of hunting seasons from 1996–2001 in the Study-Discontinuous regulation history was associated with higher growth rates compared to the Study-Continuous regulation history with consistent trend and harvest exposure results (Figs 2M, 2N and 4O). Sage-grouse populations without harvest for at least 5 years prior to 1995 (Never_1995_ and Never_1996_ regulation histories) had either relatively stable or declining trends (Fig 2A–2D and Garton et al. [3]). Compared to all other regulation histories, Never_1995_ and Never_1996_ did not consistently provide evidence of stability or increase—indicative of release from harvest pressure—after hunting was halted during this study’s timeframe nor when looking at these trends over longer timeframes from Garton et al. [3]. Thus, never hunted populations were not the most ideal reference to other regulation histories. Populations in the First-Year regulation history had opposite trajectories directly following the end of human harvest indicating lek trends were primarily influenced by local factors influencing habitat and/or weather (Fig 2E and 2F). The Permit and General regulation histories had potentially parallel interpretations where a greater proportion of a sage-grouse population exposed to permit only hunts and exposure to greater area-weighted possession limit had higher *r* (Fig 4G and 4L, respectively). However, it should be noted that years with higher *r* and greater proportion of leks subjected to permit only hunting could be considered lower harvest exposure years. Greater proportion of permit only hunting for the Permit regulation history was fall 2000 through fall 2013 with lower *r* associated with early years (1995–2000), which was when Parker Mountain and the Nevada side of Mono populations were subjected to general upland hunting regulations (Table 2). Harvest in the previous fall was negatively associated with male lek attendance the following spring in the Mono sage-grouse populations [62], which indicated additive mortality from hunting. Our most unique regulation history—Jackson—provided the possibility of observing 2 periods of population suppression from hunting (i.e., during years when hunting seasons occurred the prior fall [1995–2000 and 2002]; Fig 2G). However, the Jackson sage-grouse population started to increase 2 years prior to the first hunting season closure, and harvest exposure variables were uninformative (Table 4, Fig 2G). This raises skepticism regarding any association of the period of population growth with hunting season closures for the Jackson regulation history. Although all models for the Jackson regulation history converged and were not overly parameterized, our results from the Jackson regulation history should be treated with caution due to the small number of leks in that analysis.

Sedinger and Rotella [63] criticized the Connelly et al. [30] harvest study, because they argued that study could not determine whether harvest or density dependence influenced sage-grouse. This critique was rebutted under the premise that sage-grouse populations were unlikely to be subjected to density-dependent population regulation, because sagebrush habitat for nesting and roosting cover and as a food resource was generally not limiting [64]. More recently sage-grouse populations have been found to exhibit Gompertz and Ricker density-dependent growth [3, 13]. We also found most sage-grouse populations and regulation histories were best described with density-dependent forms of base population growth in our models (Table 4). While results from Connelly et al. [30] may have been partially confounded, our results associated with the Connelly et al. [30] harvest study were more robust after accounting for density-dependent growth. We had similar patterns of population suppression when the Study-Discontinuous was under higher harvest exposure (30-day season lengths rather than 0 or 7 days) relative to Connelly et al. [30].

Our analyses utilized commonly accepted variables that have been previously illustrated to influence adult survival and productivity; however, we were unable to decouple whether each factor influenced adult survival or reproductive rates. For example, winter precipitation during the preceding winter could be related to overwinter survival with negative [65, 66] or positive associations [36, 61, 67–70]. We chose to only examine 1- or 2-year lags of winter precipitation that would more likely be associated with reproductive rates rather than adult survival, because sage-grouse have more consistently been shown to have high overwinter survival [36, 61, 67, 68, 70, 71].

Sage-grouse populations have been known to oscillate over time, which has been attributed to weather influences on population growth [13, 72]. Greater amounts of precipitation (winter snowpack or rainfall) prior to and during the breeding season for sage-grouse have been linked to higher nest success [69, 72–74], chick survival [38, 68, 69, 75], and lek counts [13, 72]. Precipitation variables were quantified as either prior year or 2-year lag effects, which were intended to coincide with conditions promoting chick survival in previous years (Table 4). As expected, precipitation was generally positively associated with population growth regardless of time lag (Fig 4). However, we detected a couple of negative associations (e.g., Jackson and Saskatchewan) of greater amounts of precipitation resulting in lower population growth. This may be explained as high winter and spring precipitation in the Jackson sage-grouse population that may have resulted in lower recruitment of chicks in prior years or lower overwinter survival of adult sage-grouse. The negative association of SWE during winter with lek counts in Jackson may be related to lower survival of adults two winters prior to the lek count with SWE values (mean SWE_winterL1_ = 129.3 [3.2 SE]) compared to all other regulation histories (mean SWE_winterL1_ < 82.6). Whereas, PREC_springL1_ in Jackson may have been related to lower nest success and chick survival during the spring prior to the lek count with mean PREC_springL1_ = 193.2 mm (5.1 SE) compared to all other regulation histories < 147.4 mm for PREC_springL1_. Interestingly, precipitation during the summer prior to a lek count (PREC_summerL1_) in Jackson was positively connected to that lek count. In contrast, Never_1995_ regulation history had lower *r* with higher precipitation, and the negative precipitation effect was unlikely isolated extreme events rather than constantly higher precipitation values within sage-grouse populations that were in greater decline (i.e., Saskatchewan [MZ I] had higher precipitation and was in dramatic decline relative to Moses Coulee and Yakima Training [MZ VI]). However, this was not a clear pattern, because we explored the negative precipitation effects from this regulation history in greater detail by assessing an interaction between MZ and precipitation; this interaction did not fit the data well. Thus, lek trend associations with precipitation variables were likely influenced by seasonal averages rather than individual precipitation events.

Several studies have connected breeding habitat characteristics to population growth and persistence of sage-grouse leks [6, 8, 9, 12, 21, 26]. Loss of sagebrush habitat associated with environmental change or human activities has been identified as the largest threat to sage-grouse population persistence [5–7, 9]. While we have categorized predictor variables as associated with either environmental change or human activities, most of our variables that described environmental change in habitat conditions were impacted by human activities to various degrees. For example, landscapes with greater proportion and increasing proportion of cropland—a habitat-based human activity with potential of involving large landscapes—were related to decreasing *K* (Fig 5 and Table 4). Proportion of burned and forested habitat were also related to human activities with fire suppression and grazing contributing to changes in fire cycles and ultimately the size of more recent fires and the expansion of pinyon–juniper [53, 76]. Our findings of reduced *K* related to increasing proportions of burned and forested habitat between 1995–2013 were consistent with other studies assessing the effects of fire [13, 77–79] and encroaching conifers [21, 39, 80] on sage-grouse.

Much of the research assessing human factors associated with sage-grouse lek trends or persistence have found negative relationships of lek trends or persistence with human activities [6, 7, 9, 12, 24]. All human activity variables describing negative associations with *K* in our models described sagebrush habitat loss to varying degrees (Fig 5), which has been widely accepted as the primary mechanism behind declining sage-grouse populations [4, 5, 7, 9, 10, 12, 29]. While we did not detect an effect of distance to a town, distance to roads, or power line density on Λ, our specification of these variables were as stationary influencing the overall initial male count among leks. We specified these variables as stationary because the data available at large spatial scales did not allow us to construct them as annually changing variables. In reality, all of these variables were annually changing to various magnitudes throughout the western United States and southern Canada. However, it was also likely that these variables were relatively stable during 1995–2013 for much of the area within the smaller sage-grouse populations in this study. For our analyses, human population and oil and gas density variables likely served as surrogates for human-related annual habitat change in the sage-grouse populations where the stationary variables, such as roads and power lines, were changing in tandem with human population and oil and gas densities. Similar to previous research, both human population [6] and oil and gas well density [9, 12, 24, 26, 27] had negative relationships with some sage-grouse populations (Fig 5). Interestingly, the North Park population of the General regulation history was the only population with a significant oil and gas well density that did not exhibit a corresponding negative response in *K*, which could be explained by the relatively slower development pattern or other factors buffering North Park from negative consequences of oil and gas development (e.g., better placement of well pads); however, this observation deserves further assessment. Human population density was hypothesized to represent human development and disturbance corresponding to sagebrush habitat loss. Human population density was negatively associated to *K* in the Never_1995_ and Permit regulation histories but positively associated to *K* in the Study-Continuous regulation history (Fig 5). A possible explanation of the positive result may be attributed to increasing human population density in urban centers at the extreme south (near Twin Falls, Idaho, USA) of that regulation history that were not reflective of human presence or development within the interior portion of the Snake/Salmon/Beaverhead sage-grouse population.

## Conclusions

Wildlife agencies have maintained season regulations with the intention of providing sustainable hunting opportunities for sage-grouse by reducing potential for additive mortality from hunting. We found mixed results regarding the effect of hunting season regulations on population growth rate. Our best evidence for a negative effect of hunting on sage-grouse was population suppression in the Study-Continuous (Fig 2M) relative to the Study-Discontinuous (Fig 2N) from 1997–2003. Our study was the first to evaluate multiple factors influencing lek trends across time with environmental change and human activity variables being spatially and temporally explicit to each lek. This type of analysis is extremely useful to identify focal areas for conservation [81, 82]. Our population trend models will assist management agencies to better understand patterns and focus conservation efforts. As commonly thought among management agencies, not all sage-grouse populations were influenced by the same factors. However, anthropogenic, habitat, fire, and precipitation effects aligned with results from other studies. Similar to assertions by Taylor et al. [15] to focus conservation actions on increasing adult female survival, nest success, and chick survival, we suggest that sage-grouse conservation should more broadly focus on factors that increase *K*.

## Supporting information

S1 Table Descriptions and data source of variables used in open population N-mixture models assessing sage-grouse lek trends, 1995–2013. Variables were quantified within 8 km of each sage-grouse lek with the exceptions of 10 km for precipitation variables and throughout each sage-grouse population for harvest pressure variables. Lek count data were collected by states/provinces throughout the western US and southern Alberta and Saskatchewan, Canada from 1995–2013.
(DOCX)
