# Supplementary material for: Influence of environmental change, harvest exposure, and human disturbance on population trends of greater sage-grouse
Source: PLoS One. 2021 Sep 24;16(9):e0257198. doi: 10.1371/journal.pone.0257198 (PMC8462709; doi:10.1371/journal.pone.0257198)
Supplement: S1 Table — Variables were quantified within 8 km of each sage-grouse lek with the exceptions of 10 km for precipitation variables and throughout each sage-grouse population for harvest pressure variables. Lek count data were collected by states/provinces throughout the western US and southern Alberta and Saskatchewan, Canada from 1995–2013. (DOCX) [file pone.0257198.s001.docx]

**Appendix 1**

**Table S1.** Descriptions and data source of variables used in open population N-mixture models assessing sage-grouse lek trends, 1995–2013. Variables were quantified within 8 km of each sage-grouse lek with the exceptions of 10 km for precipitation variables and throughout each sage-grouse population for harvest pressure variables. Lek count data were collected by states/provinces throughout the western US and southern Alberta and Saskatchewan Canada from 1995–2013.

| Variable | Description | Resolution | Temporal component | Data source |
| --- | --- | --- | --- | --- |
| Human Activities |  |  |  |  |
| *Anthropogenic development* |  |  |  |  |
| Cropland (nCROP) | Proportion of spatial buffer classified as cropland | 30 m | Time step addition or subtraction | National Landcover Datatset (NLCD; 1992, 2001, 2006, 2011; Homer et al. 2012) |
| Cropland (sCROP) | Proportion of spatial buffer classified as cropland | 300 m | Time step addition or subtraction | ESA Climate Change Initiative (SPOT; 2000, 2005, 2010) |
| Human population (HUM) | Annual density (no./km^2^) quantified by US or provincial county | County | Annual addition or subtraction | US Census Bureau and Canadian CANSIM (1995–2013) |
| Oil and gas (OIL) | Density of oil and gas wells (no./km^2^)^a^ | Point | Annual addition or subtraction | State oil and gas commissions (1990–2013) |
| Town distance | Average distance to a town >10,000 people | 30 m | Stationary | US Baruch Geoportal and Canada AltaLIS and GeoSask |
| Power lines | Density of transmission lines | Line | Stationary | US Energy Information Administration and Canada AltaLIS |
| Roads and rail | Density of roads and railroads (km/km^2^) | Line | Stationary | US Census Bureau TIGER and Statistics Canada Road Network |
| Wind power | Density of wind turbines (km/km^2^)^a^ | Point | Annual addition or subtraction | US Geological Survey and Natural Resources Canada Wind Resource (1990–2013) |
| *Harvest Pressure* |  |  |  |  |
| Harvest area (HAREA) | Proportion of area within sage-grouse population open to legal hunting | Sage-grouse population | Annual addition or subtraction | State/provincial governments (1995–2013) |
| Bag/possession (BAG) | Area weighted average bag and possession limits | Sage-grouse population | Annual change | State/provincial governments (1995–2013) |
| Season length (S_LEN) | Area weighted average season length | Sage-grouse population | Annual change | State/provincial governments (1995–2013) |
| Area open to permit hunting (PERMIT) | Proportion of sage-grouse population open to permit only hunting | Sage-grouse population | Annual change | State/provincial governments (1995–2013) |
| Environmental Change |  |  |  |  |
| *Habitat* |  |  |  |  |
| Forested habitat (nTREE) | Proportion of all conifer-dominated landcover types | 30 m | Time step addition or subtraction | NLCD (1992, 2001, 2006, 2011; Homer et al. 2012) |
| Forested habitat  (sTREE) | Proportion of all conifer-dominated landcover types | 300 m | Time step addition or subtraction | SPOT (2000, 2005, 2010) |
| Burned area (FIRE) | Proportion of area burnt within 8 km of a lek^b^ | 30 m | Annually cumulative | Monitoring Trends in Burn Severity (MTBS; 1990–2013; Eidenshink et al. 2007) |
| *Precipitation* |  |  |  |  |
| Winter precipitation (PREC_winter_) | Total precipitation December–February (mm) within 10 km^b^ | 1,000 m | Annual change | Daymet (1994–2013; Thornton et al. 1997, Thornton et al. 2014) |
| Spring precipitation (PREC_spring_) | Total precipitation (mm) March–May within 10 km^b^ | 1,000 m | Annual change | Daymet (1994–2013) |
| Summer precipitation (PREC_summer_) | Total precipitation (mm) July–August within 10 km^b^ | 1,000 m | Annual change | Daymet (1994–2013; Thornton et al. 1997, Thornton et al. 2014) |
| Winter snow water equivalent (SWE_winter_) | SWE (kg/m^2^) December–February within 10 km^b^ | 1,000 m | Annual change | Daymet (1994–2013; Thornton et al. 1997, Thornton et al. 2014) |
| Spring SWE (SWE_spring_) | SWE (kg/m^2^) March–May within 10 km^b^ | 1,000 m | Annual change | Daymet (1994–2013; Thornton et al. 1997, Thornton et al. 2014) |

^a^Quantified as value during previous season, three seasons prior (lag3), or five seasons prior (lag5) to lek count.

^b^Quantified as value during previous season (lag1) or two seasons (lag2) prior to lek count.
